# Engineering of *Escherichia coli* for Krebs cycle-dependent production of malic acid

**DOI:** 10.1186/s12934-018-0959-y

**Published:** 2018-07-16

**Authors:** Debora Trichez, Clément Auriol, Audrey Baylac, Romain Irague, Clémentine Dressaire, Marc Carnicer-Heras, Stéphanie Heux, Jean Marie François, Thomas Walther

**Affiliations:** 1 LISBP, Université de Toulouse, CNRS, INRA, INSA, Toulouse, France; 2 TWB, 3 rue Ariane, 31520 Ramonville-St. Agnes, France; 3 Present Address: Cinabio, Cinabio-Adisseo France S.A.S., 31077 Toulouse, France; 4 Present Address: Institute of Natural Materials Technology, Technische Universität Dresden, 01062 Dresden, Germany

**Keywords:** Malic acid, *Escherichia coli*, Flux analysis, Metabolic engineering

## Abstract

**Background:**

Malate is a C4-dicarboxylic acid widely used as an acidulant in the food and beverage industry. Rational engineering has been performed in the past for the development of microbial strains capable of efficient production of this metabolite. However, as malate can be a precursor for specialty chemicals, such as 2,4-dihydroxybutyric acid, that require additional cofactors NADP(H) and ATP, we set out to reengineer *Escherichia coli* for Krebs cycle-dependent production of malic acid that can satisfy these requirements.

**Results:**

We found that significant malate production required at least simultaneous deletion of all malic enzymes and dehydrogenases, and concomitant expression of a malate-insensitive PEP carboxylase. Metabolic flux analysis using ^13^C-labeled glucose indicated that malate-producing strains had a very high flux over the glyoxylate shunt with almost no flux passing through the isocitrate dehydrogenase reaction. The highest malate yield of 0.82 mol/mol was obtained with *E. coli Δmdh Δmqo ΔmaeAB ΔiclR ΔarcA* which expressed malate-insensitive PEP carboxylase Ppc^K620S^ and NADH-insensitive citrate synthase GltA^R164L^. We also showed that inactivation of the dicarboxylic acid transporter DcuA strongly reduced malate production arguing for a pivotal role of this permease in malate export.

**Conclusions:**

Since more NAD(P)H and ATP cofactors are generated in the Krebs cycle-dependent malate production when compared to pathways which depend on the function of anaplerotic PEP carboxylase or PEP carboxykinase enzymes, the engineered strain developed in this study can serve as a platform to increase biosynthesis of malate-derived metabolites such as 2,4-dihydroxybutyric acid.

**Electronic supplementary material:**

The online version of this article (10.1186/s12934-018-0959-y) contains supplementary material, which is available to authorized users.

## Background

The four-carbon dicarboxylic acid malate is a Krebs cycle intermediate and a compound of considerable economic interest. It is mainly used as an acidulant in the food and beverage industry, or as a precursor for specialty chemicals [1, 2].

Currently, d/l-malic acid is chemically produced by hydration of maleic anhydride and has an estimated annual market volume of ~ 10 kt/year. However, due to its potential to replace maleic acid as a major building block in the chemical industry, its annual production could rise to ~ 200 kt if a cost-efficient biochemical production process could be developed [2]. Therefore, malic acid has been ranked as one of the 12 top target molecules of biochemical production processes [3], and much research effort has been invested in recent years to optimize the metabolic network and cultivation conditions of malate-producing organisms.

The filamentous fungus *Aspergillus flavus* was the first identified natural malate-producing organism [4]. After optimization of cultivation conditions, it accumulated 113 g/l malate with a yield and productivity of 1.26 mol/mol and 0.59 g/(lh), respectively [5]. However, since *A. flavus* produces toxic aflatoxin, this organism was never employed in industrial production processes. During the search for alternative natural malate-producing organisms, the yeast *Zygosaccharomyces rouxii* and the filamentous fungi *Aspergillus oryzae* and *Penicillium sclerotiorum* were shown to accumulate malate to high levels, reaching 92 g/l at a yield of 1.2 mol/mol for the latter organism [6–8].

In complementary studies carried out in yeast, bacteria, and fungi, it was tried to further increase malate production by rational optimization of the metabolic network. The oxaloacetate (OAA)-yielding carboxylation of either phospho*enol*pyruvate (PEP) or pyruvate, followed by direct reduction of OAA to produce malate represents the most efficient metabolic pathway, which has a theoretical yield of 2 mol malate per mol glucose [9]. Therefore, all metabolic engineering endeavors to increase malate production focused on the optimization of this pathway. The overexpression of pyruvate carboxylase, malate dehydrogenase, and malate permease activities in a pyruvate-overproducing *Saccharomyces cerevisiae* platform strain enabled the accumulation of 59 g/l malate at a yield of 0.42 mol/mol [9–11]. Using a similar strategy, malate production by an *A. oryzae* strain could be increased to 154 g/l and a yield of 1.38 mol/mol after overexpressing pyruvate carboxylase, and a malate permease [6]. While the optimal function of the processes employing yeast and fungi required aerobic cultivation conditions, anaerobic malate production was demonstrated with *Escherichia coli.* A strain devoid of all alternative fermentative pathways and evolved for the production of succinate [12, 13] was converted into a malate producer by additionally deleting the genes coding for malic enzyme, fumarate reductase and fumarase activities. The strain accumulated 34 g/l malate with a yield and productivity of 1.42 mol/mol and 0.47 g/(l h), respectively [14]. Malate and succinate production in the evolved strain relied on mutations that conferred increased expression of PEP carboxykinase (Pck) and inactivation of the PTS sugar uptake system [15, 16]. Together, these modifications enabled an ATP-producing carboxylation of PEP and thus increases the energy output of the pathway. The microbial production of malic acid by the use of additional microorganisms and alternative substrates was discussed in detail in two recent reviews [17, 18].

The biosynthesis of malate from glucose via direct reduction of oxaloacetate is redox neutral and produces a maximum of two mole ATP per glucose molecule if PEP is converted into oxaloacetate via the ATP-forming Pck. Thus, in biosyntheses that employ additional reduction steps for the conversion of malate into other target molecules, cofactor supply may become limiting. We recently proposed a synthetic pathway which converts malate into DHB requiring one mol of ATP and two moles of NAD(P)H [19]. These additional cofactors cannot be produced by employing the above-cited conventional pathways for malate biosynthesis. Therefore, we set out to investigate metabolic engineering requirements to enable aerobic malate production via Krebs cycle and glyoxylate shunt, which increases ATP and NADH production at the expense of a decreased maximum malate yield which drops to 1.33 mol/mol [9].

We show that significant malate production through the Krebs cycle could not be achieved by solely deleting all malate dehydrogenases and malic enzymes, but additionally required the over-expression of a malate-insensitive PEP carboxylase mutant. Malate production was further increased by the inactivation of the acetate pathways, or by the overexpression of a NADH-insensitive citrate synthase mutant. Inactivation of the dicarboxylic acid transporter DcuA strongly reduced malate production arguing for a pivotal role of this permease in malate export. The best strain produced malate from glucose at a yield of 0.82 mol/mol.

## Methods

### Media and cultivation conditions

Strains were grown in Luria–Bertani (LB) broth (containing per liter: 10 g tryptone, 5 g yeast extract, 10 g NaCl, and 50 µg kanamycin sulfate and 25 µg chloramphenicol when necessary) [49] during strain construction and in starter cultures. All growth and pre-cultures were carried out in defined mineral medium. One liter mineral medium contained 10 g glucose, 18 g Na_2_HPO_4_ * 12 H_2_O, 3 g KH_2_PO_4_, 0.5 g NaCl, 2 g NH_4_Cl, 0.5 g MgSO_4_ * 7 H_2_O, 0.015 CaCl_2_ * 2 H_2_O, 1 ml of 0.06 mol/l FeCl_3_ stock solution (prepared in 100 times diluted concentrated HCl), 2 ml of 10 mM thiamine HCl stock solution, 20 g MOPS, 50 µg kanamycin sulfate (and 25 µg chloramphenicol when necessary), and 1 ml of trace element solution (containing per liter: 0.4 g Na_2_EDTA * 2H_2_O, 1.8 g CoCl_2_ * 6 H_2_O, 1.8 g ZnSO_4_ * 7 H_2_O, 0.4 g Na_2_MoO_4_ * 2 H_2_O, 0.1 g H_3_BO_3_, 1.2 g MnSO_4_ * H_2_O, 1.2 g CuCl_2_ * H_2_O). Medium was adjusted to pH 7 and filter-sterilized. Pre-cultures were inoculated at OD of ~ 0.5 from starter cultures that were grown overnight in LB. Growth cultures were inoculated at OD of ~ 0.2 from exponentially growing pre-cultures. When OD of the growth cultures reached 0.6, 1 mM isopropyl β-d-1-thiogalactopyranoside (IPTG) was added. Strains were incubated in 250 ml flasks containing 30 ml medium, and shaken at 200 rpm and 37 °C in a rotary shaker (Infors). Samples were regularly withdrawn to follow growth and metabolite secretion.

### Construction of strains and plasmids

#### Strain construction

All strains were derived from the *E. coli* K-12 substr. MG1655 wild-type strain. Gene deletions were introduced successively using the phage transduction method adapted from Miller [50]. Strains carrying the desired single deletions were recovered from the Keio collection [51]. Phage lysates of single deletion mutants were prepared by inoculating 10 ml of LB medium containing 50 µg/ml kanamycin, 2 g/l glucose, and 5 mM CaCl_2_ with 100 µl of overnight precultures. Following an incubation of 1 h at 37 °C, 200 µl of phage lysate prepared from the wild-type *E. coli* K-12 MG1655 strain were added, and cultures were incubated for another 2–3 h until cell lysis had completed. After addition of 200 µl chloroform, cell preparations were first vigorously vortexed and then centrifuged for 10 min at 4500×*g*. The clear lysate was recovered and stored at 4 °C. The receptor strain was prepared for phage transduction by an overnight cultivation at 37 °C in LB medium. A volume of 1.5 ml of the preculture was centrifuged at 1500×*g* for 10 min. The supernatant was discarded and the cell pellet was resuspended in 600 µl of a solution containing 10 mM MgSO_4_ and 5 mM CaCl_2_. The transduction was carried out by mixing 100 µl of the solution containing the receptor strain with 100 µl of lysate and incubating this mixture at 30 °C for 30 min. Thereafter, 100 µl of a 1 M sodium citrate solution were added followed by vigorous vortexing. After addition of 1 ml LB medium, the cell suspension was incubated at 37 °C for 1 h before spreading the cells on LB agar dishes containing 50 µg/ml kanamycin. Clones able to grow in presence of the antibiotic were confirmed by colony PCR to contain the desired deletion using the primers listed in Table 3. The resistance gene (FRT-kan-FRT) was subsequently excised from the chromosome using the FLP recombinase-harbouring plasmid pCP20 [52] leaving a scar region containing one FRT site. Kanamycin resistant mutants were transformed with pCP20, and ampicillin-resistant transformants were selected at 30 °C. Transformants were then grown on solid LB medium at 37 °C and tested for loss of all antibiotic resistances. Excision of the FRT-kanamycin cassette was analyzed by colony PCR using crimson taq polymerase and the flanking locus-specific primers (Table 1). All strains used in this study are listed in Table 2.

**Table 1:** Primers and plasmids used in this study

Primers	Relevant characteristics	Source
ppc_clon_for	TATAATCCCGGGATGAACGAACAATATTCC	This study
ppc_clon_for	TATAATTCTAGATTAGCCGGTATTACGCAT	This study
ppc_k620s_for	CGCTTTAGCTATGGTCTGCCAGAAATCACCGAG	This study
ppc_k620s_rev	CCATAGCTAAAGCGGATCATCTCGCCC	This study
ppc_sRBS_for	AACAGAATTCGAGCTCGGTACCCGGGGTTTAACTTTAAGAAGGAGATATACCATGAACGAACAATATTCCGCATTGCGTAGTAATG	This study
ppc_sRBS_rev	TATAATTCTAGAATTAGCCGGTATTACGCATACC	This study
gltA_clon_for	TATATAGAGCTCATGGCTGATACAAAAGCAAAACTCACC	This study
gltA_clon_rev	TATAATAAGCTTTTAACGCTTGATATCGCTTTTAAAGTCGC	This study
gltA_R164L_for	ATTGCCGCGTTCCTCCTGCTGTCGAAAATGCCGACTATGGCCGCG	This study
gltA_R164L_rev	CGCGGCCATAGTCGGCATTTTCGACAGCAGGAGGAACGCGGCAAT	This study
gltA_clon_for_1	TGCGTAATACCGGCTAAAGGAGGAACCGTATGGCTGATACAAAAGCAAAACTC	This study
gltA_clon_rev_1	CATCCGCCAAAACAGAAGCTTTTAACGCTTGATATCGCTTTTAAAG	This study
Plasmids		
pKD4	amp	[53]
pKD46	amp	[53]
pCP20	amp cho	[53]
pEXT20	amp	[28]
pACT3	cho	[28]
pACT3w-ppc_wt_	pACT3 expressing wild-type *ppc* gene from *E. coli* from weak RBS	This study
pACT3w-ppc_K620S_	pACT3 expressing *ppc*_*K620S*_ mutant gene from *E. coli* from weak RBS	This study
pEXT20w-ppc_K620S_	pEXT20 expressing *ppc*_*K620S*_ mutant gene from *E. coli* from strong RBS	This study
pACT3s-ppc_K620S_	pACT3 expressing *ppc*_*K620S*_ mutant gene from *E. coli* from strong RBS	This study
pACT3w-ppc_K620S_-gltA_R164L_	pACT3w-ppc_K620S_ expressing *gltA*_*R164L*_ mutant gene from *E. coli*	This study

**Table 2:** *Escherichia coli* strains used in this study

Strains	Relevant characteristics	Source
Wild-type	*E. coli* K-12 MG1655 F^−^ λ^−^ ilvG- rfb-50 rph-1	ATCC47076
Keio strains	F-, *Δ(araD*-*araB)567*, *ΔlacZ4787(::rrnB*-*3)*, *λ*-, *rph*-*1*, *Δ(rhaD*-*rhaB)568*, *hsdR514* carrying single gene deletions	[54]
Sy62	Δ*mdh*::FRT	This study
Sy111	Δ*mqo*::FRT	This study
Sy68	Δ*mdh*::FRT Δ*mqo*::FRT	This study
Sy102	Δ*mdh*::FRT Δ*mqo*::FRT Δ(*ackA*-*pta)*::FRT	This study
Sy161	Δ*mdh*::FRT Δ*mqo*::FRT Δ(*ackA*-*pta)*::FRT Δ*maeB*::FRT	This study
Sy162	Δ*mdh*::FRT Δ*mqo*::FRT Δ(*ackA*-*pta)*::FRT Δ*maeA*::FRT	This study
Sy168	Δ*mdh*::FRT Δ*mqo*::FRT Δ(*ackA*-*pta)*::FRT Δ*maeA*::FRT Δ*maeB*::FRT	This study
Sy249	Sy68 expressing pACT3w-ppc_K620S_	This study
Sy242	Sy102 expressing pACT3w-ppc_K620S_	This study
Sy252	Sy161 expressing pACT3w-ppc_K620S_	This study
Sy254	Sy162 expressing pACT3w-ppc_K620S_	This study
Sy280	Sy168 expressing pACT3w-ppc_K620S_	This study
Sy279	Sy168 expressing pACT3w-ppc_wt_	This study
Sy320	Sy168 expressing pEXT20 s-ppc_K620S_	This study
Sy321	Sy168 expressing pEXT20w-ppc_K620S_	This study
Sy322	Sy168 expressing pACT3 s-ppc_K620S_	This study
Sy502	Δ*mdh*::FRT Δ*mqo*::FRT Δ*maeA*::FRT Δ*maeB*::FRT expressing pACT3w-ppc_K620S_	This study
Sy504	Δ*mdh*::FRT Δ*mqo*::FRT Δ*maeA*::FRT Δ*maeB*::FRT Δ*iclR*::FRT expressing pACT3w-ppc_K620S_	This study
Sy510	Δ*mdh*::FRT Δ*mqo*::FRT Δ*maeA*::FRT Δ*maeB*::FRT Δ*iclR*::FRT Δ*ackA*::FRT expressing pACT3w-ppc_K620S_	This study
Sy512	Δ*mdh*::FRT Δ*mqo*::FRT Δ*maeA*::FRT Δ*maeB*::FRT Δ*iclR*::FRT Δ*ackA*::FRT Δ*poxB*::FRT expressing pACT3w-ppc_K620S_	This study
Sy729	Δ*mdh*::FRT Δ*mqo*::FRT Δ*maeA*::FRT Δ*maeB*::FRT Δ*iclR*::FRT Δ*arcA*::FRT expressing pACT3w-ppc_K620S_	This study
Sy731	Δ*mdh*::FRT Δ*mqo*::FRT Δ*maeA*::FRT Δ*maeB*::FRT Δ*iclR*::FRT Δ*arcA*::FRT expressing pACT3w-ppc_K620S_-gltA_R163L_	This study
Sy936	Δ*mdh*::FRT Δ*mqo*::FRT Δ*maeA*::FRT Δ*maeB*::FRT Δ*iclR*::FRT Δ*arcA*::FRT Δ*edd*-*eda*::FRT expressing pACT3w-ppc_K620S_-gltA_R163L_	This study
Sy939	Δ*mdh*::FRT Δ*mqo*::FRT Δ*maeA*::FRT Δ*maeB*::FRT Δ*iclR*::FRT Δ*arcA*::FRT Δ*edd*-*eda*::FRT Δ*aspA*::FRT expressing pACT3w-ppc_K620S_-gltA_R163L_	This study

#### Plasmid construction

The wild-type *ppc* gene was PCR amplified from genomic DNA the *E. coli* MG1655 strain using Phusion polymerase (Thermo-Scientific) and the forward and reverse primers ppc_clon_for and ppc_clon_frev respectively. The resulting DNA fragment was ligated into pACT3 [26] using *Sma*I and *Xba*I restriction sites to obtain vector pACT3w-ppcwt. The amino acid exchange Lys620Ser was introduced into ppc by site directed PCR mutagenesis using primers ppc_k620s_for and ppc_k620s_rev. The PCR product was *Dpn*I digested and transformed into NEB 5-alpha competent *E. coli* cells (NEB). The resulting plasmid, pACT3w-ppcK620S was isolated and verified by DNA sequencing to contain the desired mutation. Subcloning into the pEXT20 vector was achieved using *Sal*I and *Kpn*I restriction sites. To replace the original ribosome-binding site (RBS) of pACT3 in front of the ppcK620S gene by a stronger one, ppcK620S was PCR amplified using primers ppc_sRBS_for and ppc_sRBS_for, respectively, and cloned into the pACT3 and pEXT20 vectors using *Sma*I and *Xba*I restriction sites. The wild-type gltA gene was PCR-amplified from genomic DNA of the *E. coli* MG1655 strain using pfu polymerase primers gltA_clon_for and gltA_clon_rev. The resulting DNA fragment was cloned into the pACT3 vector using *Sac*I and *Hin*dIII restriction sites. The amino acid exchange R164L was introduced into the gltA gene by site directed PCR mutagenesis using primers gltA_R164L_for and gltA_R164L_rev. The PCR product was *Dpn*I digested and transformed into NEB 5-alpha competent *E. coli* cells (NEB). The resulting plasmid, pACT3-gltAR164L was isolated and verified by DNA sequencing to contain the desired mutation. For construction of vector pACT3w-ppcK620S-gltAR164L, the mutant gltAR164L gene was amplified from the pACT3-gltAR164L plasmid using Phusion polymerase (Biolabs) and primers gltA_R164L_for_1 and gltA_R164L_for_2. The PCR product was purified and recombined into the pACT3w-ppcK620S vector that was linearized with *Hin*dIII and *Xba*I, using the In-Fusion^®^ HD Cloning Kit (Clontech).

### Analytical techniques

Glucose and extracellular metabolites were quantified on a Dionex Ultimate 3000 HPLC system equipped with a UV and an RI (Shimadzu RID 10A) detector, an Aminex HPX-87H column thermostated at 32 °C, and a “Micro-Guard cation H Refill Cartridge” pre-column. Dilute sulfuric acid (1.25 mM) was used as the mobile phase at an isocratic flow rate of 0.5 ml/min.

### Metabolic flux analysis

#### Culture conditions

The strain was grown in batch culture using a 0.5 l fermenter (Multifors Infors HT, The Netherlands) with a working volume of 0.4 l coupled to a Mass Gas Analyzer (Proline Dycor, Ametek process instrument, USA). Cells were cultivated in modified M9 medium adapted for IC-MS/MS analysis [31] that contained 50 mM glucose (ratio of uniformly labeled to C1-labeled glucose was 1/4), 0.6 mM IPTG and 25 µg/ml chloramphenicol. Temperature, pH, air flow, and stirring speed were maintained at 37 °C, pH 7 (with KOH 1 M), 0.2 l/min and 800 rpm, respectively. Under these conditions, oxygen concentration remained above 50% of saturation throughout the cultivation. The N_2_, O_2_, Argon, CO_2_ concentrations in the bioreactors off-gas were measured on-line using a Mass Gas Analyzer.

#### Quantification of extracellular metabolites

Culture broth was separated from cells by centrifugation over 0.45 µm pore size membranes (Minisart, Sartorius) and stored at − 20 °C until analysis. To quantify secreted metabolites, clear supernatant was subject to 1H 1D-NMR analysis (Avance 800 MHz spectrometer, Bruker, Germany) carried out at 292 °K, using a 30° pulse and a relaxation delay of 20 s. Trimethylsilyl propionate was used as internal standard.

#### Quantification of mass isotopologues in intracellular metabolites

Approximately 0.8 ml samples (cells + culture medium) with a cell concentration of 0.4 g/l were manually withdrawn from the bioreactor and directly quenched and extracted by mixing with 3 ml of a mixture of acetonitrile, methanol, and formic acid (0.1 M) (40:40:20 v/v), and incubation at − 20 °C for 1 h. Next, the tubes were moved to − 80 °C until further treatment. Sample evaporation was performed in a rotavapor (Büchi, Switzerland) during approximately 15 h until complete dryness. Afterwards, the samples were dissolved in a 500 μl ultrapure water and stored at − 20 °C until analysis. IC-MS/MS analysis was carried out as described by [34] to quantify the isotopic enrichment of selected metabolites. The analyzed metabolites were PEP, R5P, S7P, 6PG, G6P, FBP, F6P, 23 PG, Cit, Fum, Mal and Suc. Raw peak areas were corrected for the contribution of all naturally abundant isotopes using the IsoCor software [53]. In addition, label incorporation into secreted acetate and malate was measured using 1H 1D-NMR as described above.

#### Calculation of fluxes

Intracellular carbon fluxes were calculated from data on mass isotopologue enrichments and production/consumption rates of extracellular metabolites using the Influx_s software [21].

## Results

### Efficient malate production requires inactivation of malic enzymes and dehydrogenases, and appropriate expression of malate-insensitive PEP carboxylase

**Fig. 1:**
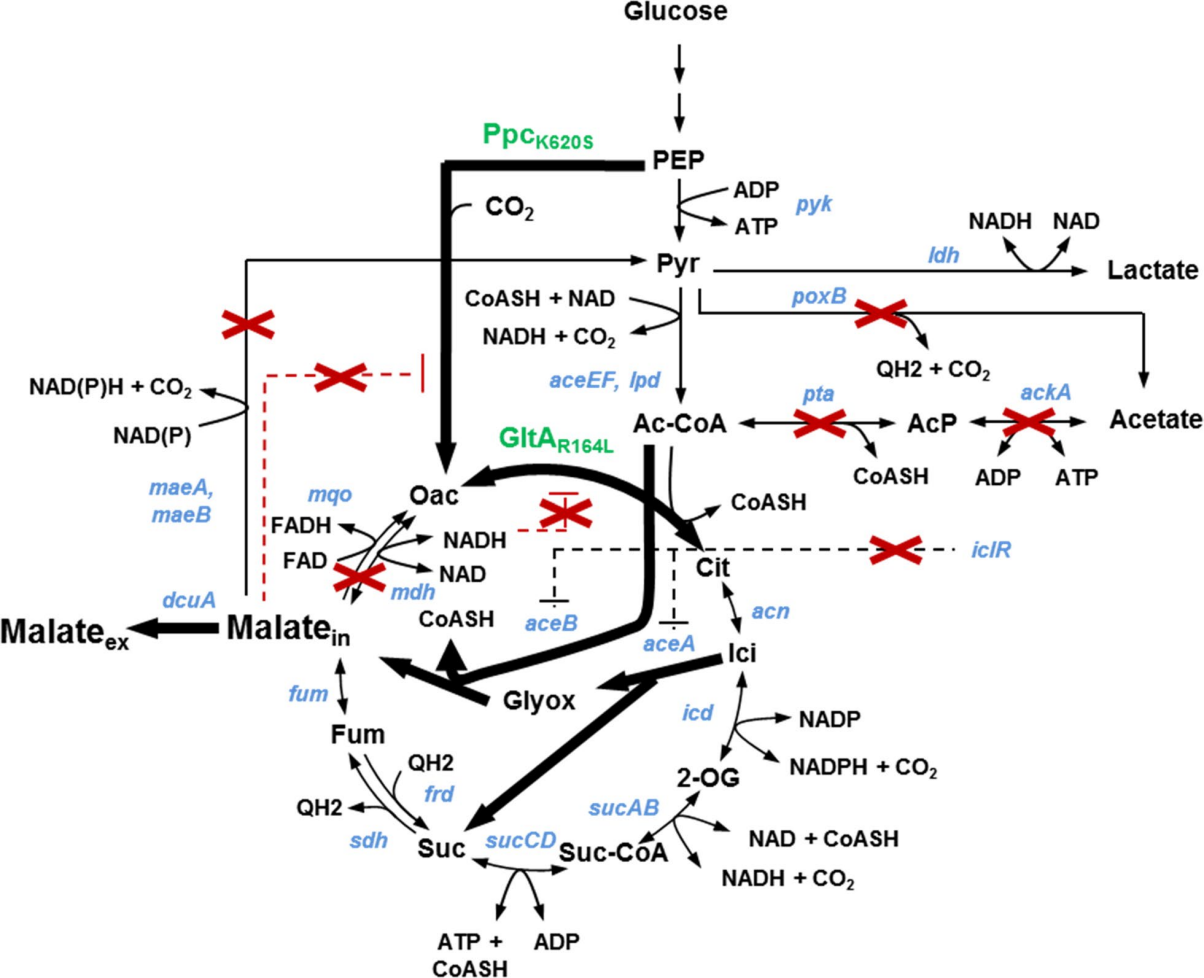
Relevant metabolic pathways during aerobic production of malate *in Escherichia coli*. Gene names are depicted in blue, names of enzymes that were overexpressed to alleviate allosteric inhibition are depicted in green. Bold lines indicate enzymatic activities that were overexpressed or derepressed, dashed black lines indicate transcriptional repression, and dashed red lines indicate allosteric inhibition. Red crosses indicate deletions of genes and attenuated allosteric inhibitions (*Oac* oxaloacetate, *Cit* citrate, *Ici* isocitrate, *2-OG* 2-oxoglutarate, *Suc-CoA* Succinyl-CoA, *Suc* succinate, *Pyr* pyruvate, *Ac-CoA* acetyl-CoA, *Glyox* glyoxylate)

To investigate the Krebs cycle-dependent malate production, we step-wise introduced deletions in competing metabolic pathways (Fig. 1) and analyzed the behavior of the mutants in shake flask cultures using M9 mineral medium which contained 10 g/l glucose as a carbon source. To trigger malate secretion under aerobic conditions we first interrupted the Krebs cycle by deleting the soluble and membrane associated malate dehydrogenases, encoded by *mdh* and *mqo*, respectively [20, 21]. However, no significant malate accumulation in the culture medium was observed in the single and double mutants but acetate production strongly increased (Table 1). We therefore additionally deleted the acetate-forming pathway, encoded by *ackA*-*pta* [22], and the two malic enzymes, encoded by *maeA* and *maeB*. The resulting quintuple mutant Sy162 produced malate at a molar yield of 0.12 (Table 3).

**Table 3:** Fermentation products of *E. coli* MG1655 mutants in mineral medium with glucose as the carbon source

Strain	Genotype	Plasmid	Cells (g/g)	Malate (mol/mol)	Fumarate (mol/mol)	Pyruvate (mol/mol)	Acetate (mol/mol)
*K12*	Wild-type	–	0.32 ± 0.02	0.01 ± 0.01	0.00	0.28 ± 0.10	0.48 ± 0.04
*Sy62*	*Δmdh*	–	0.27 ± 0.02	0.06 ± 0.00	0.00	0.17 ± 0.02	0.88 ± 0.15
*Sy111*	*Δmqo*	–	0.33 ± 0.03	0.06 ± 0.03	0.00	0.18 ± 0.01	0.34 ± 0.08
*Sy68*	*Δmdh Δmqo*	–	0.27 ± 0.02	0.04 ± 0.00	0.00	0.15 ± 0.02	1.31 ± 0.20
*Sy102*	*Δmdh Δmqo ΔackA*-*pta*	–	0.23 ± 0.02	0.01 ± 0.00	0.00	1.30 ± 0.05	0.21 ± 0.02
*Sy162*	*Δmdh Δmqo ΔackA*-*pta ΔmaeA*	–	0.22 ± 0.02	0.03 ± 0.02	0.00	0.17 ± 0.02	0.79 ± 0.02
*Sy168*	*Δmdh Δmqo ΔackA*-*pta ΔmaeA ΔmaeB*	–	0.16 ± 0.06	0.12 ± 0.04	0.06 ± 0.02	0.24 ± 0.04	1.06 ± 0.25
Sy279	*Δmdh Δmqo ΔackA*-*pta ΔmaeA ΔmaeB*	pACT3w-ppc_wt_	0.12 ± 0.03	0.13 ± 0.02	0.04 ± 0.01	0.95 ± 0.02	0.69 ± 0.10
*Sy320*	*Δmdh Δmqo ΔackA*-*pta ΔmaeA ΔmaeB*	pEXT20s-ppc_K620S_	0.18 ± 0.01	0.23 ± 0.01	0.03 ± 0.01	0.06 ± 0.03	1.65 ± 0.09
*Sy321*	*Δmdh Δmqo ΔackA*-*pta ΔmaeA ΔmaeB*	pEXT20w-ppc_K620S_	0.13 ± 0.01	0.14 ± 0.03	0.05 ± 0.04	0.96 ± 0.03	0.77 ± 0.08
*Sy322*	*Δmdh Δmqo ΔackA*-*pta ΔmaeA ΔmaeB*	pACT3s-ppc_K620S_	0.28 ± 0.02	0.37 ± 0.02	0.14 ± 0.01	0.00 ± 0.00	0.64 ± 0.01
*Sy280*	*Δmdh Δmqo ΔackA*-*pta ΔmaeA ΔmaeB*	pACT3w-ppc_K620S_	0.21 ± 0.00	0.48 ± 0.02	0.11 ± 0.02	0.28 ± 0.05	0.33 ± 0.04

Data is presented as means ± STDV of at least three replicate experiments

It was previously demonstrated that the aerobic production of the Krebs cycle intermediates succinate and fumarate could be improved by increasing expression of the anaplerotic enzymes phosphoenolpyruvate (PEP) carboxylase (Ppc) [23, 24], or pyruvate carboxylase [25]. However, overexpression of the natural Ppc from *E. coli* did not increase malate production (Table 1). We therefore hypothesized that intracellular accumulation of malate inhibits Ppc [26], thereby preventing production of oxaloacetate and thus synthesis of the first Krebs cycle intermediate citrate. We alleviated malate feedback inhibition by introducing mutation Lys620Ser into the Ppc enzyme [27] and varied the expression level of the resulting mutant using the high and medium copy plasmids, pEXT20 and pACT3 [28], respectively, and strong and weak ribosome binging sites (RBS). Indeed, we found that the expression of the Ppc_K620S_ mutant strongly improved malate production, and that malate yield varied by more than threefold depending on the choice of the expression vector and strength of the RBS. Our data does not provide a clear explanation for the observed differences in the production of malate and other metabolic by-products, since they can be due to changes in the activity of the Ppc_K620S_ enzyme or due to the altered metabolic burden imposed by different plasmid copy numbers. However, it was previously demonstrated by Lee et al. [29] that efficient production of threonine required moderate over-expression of Ppc and that an excessively high Ppc activity was deleterious for the production of this amino acid. In line with these results, we found that the medium-copy plasmid pACT3w-ppc_K620S_, which contained a weak RBS and which can therefore be considered the weakest expression system of the four tested vectors, produced the highest malate yield of 0.48 mol/mol (Table 1). Plasmid pACT3w-ppc_K620S_ was therefore used in all further experiments (Table 4).

**Table 4:** Fermentation products of *E. coli* mutants in mineral medium with glucose as the carbon source

Strain	Additional genotype modifications	Plasmid	Cells (g/g)	Malate (mol/mol)	Fumarate (mol/mol)	Pyruvate (mol/mol)	Acetate (mol/mol)
Sy502	–	pACT3w-ppc_K620S_	0.20 ± 0.06	0.40 ± 0.08	0.09 ± 0.03	0.00	0.56 ± 0.03
Sy504	*ΔiclR*	pACT3w-ppc_K620S_	0.19 ± 0.05	0.42 ± 0.05	0.10 ± 0.04	0.00	0.55 ± 0.06
Sy510	*ΔiclR ΔackA*	pACT3w-ppc_K620S_	0.21 ± 0.01	0.55 ± 0.02	0.11 ± 0.01	0.01 ± 0.00	0.43 ± 0.00
Sy508	*ΔiclR ΔpoxB*	pACT3w-ppc_K620S_	0.16 ± 0.02	0.30 ± 0.07	0.07 ± 0.03	0.11 ± 0.02	0.29 ± 0.22
Sy512	*ΔiclR ΔackA ΔpoxB*	pACT3w-ppc_K620S_	0.22 ± 0.03	0.35 ± 0.00	0.10 ± 0.01	0.56 ± 0.01	0.02 ± 0.00
Sy729	*ΔiclR ΔarcA*	pACT3w-ppc_K620S_	0.15 ± 0.02	0.53 ± 0.03	0.06 ± 0.04	0.04 ± 0.03	0.38 ± 0.04
Sy731	*ΔiclR ΔarcA*	pACT3w-ppc_K620S_-gltA_R164L_	0.15 ± 0.02	0.82 ± 0.07	0.06 ± 0.02	0.02 ± 0.04	0.25 ± 0.09

All strains are derived from Sy502 strain (MG1655 *Δmdh Δmqo ΔmaeA ΔmaeB)*

Data is presented as means ± STDV of at least three replicate experiments

We then tested the impact of expressing the malate-insensitive Ppc_K620S_ mutant in strains that were still wild-type for at least one malic enzyme, or that still contained the functional AckA-Pta-dependent acetate pathway. We found that malate yields strongly decreased in strains expressing at least one malic enzyme and did not exceed 0.15 mol/mol (Additional file 1: Table S1), whereas the presence of the AckA-Pta pathway only had a minor impact on the malate yield which was estimated at 0.4 mol/mol (Table 2). Taken together these results show that inactivation of all malate dehydrogenases and malic enzymes together with appropriate expression of a malate insensitive Ppc mutant are necessary and sufficient to enable significant malate production.

### Characterization of carbon flux distribution in a malate-producing strain and further genotype optimization

After having identified the major requirements for aerobic malate production in *E. coli*, we carried out a detailed analysis on the impact of additional genetic modifications on malate production by this organism. Starting from strain Sy502, which carried deletions in both malate dehydrogenases and malic enzymes (*Δmdh Δmqo ΔmaeA ΔmaeB*) and which had a malate yield of 0.4 mol/mol, we sequentially deleted the transcriptional repressor of the glyoxylate shunt genes (*aceA*, *aceB*), encoded by *iclR* [30, 31], and the enzymes acetate kinase and pyruvate oxidase, encoded by *ackA* [32] and *poxB* [33], respectively. We observed that the transcriptional derepression of the glyoxylate pathway upon deletion of *iclR* in strain Sy502 giving rise to strain Sy504 did not result in an increased malate yield. In addition, acetate production remained unchanged when compared to strain Sy502 (Table 2), which is at variance to the behavior of an *ΔiclR* single mutant that is known to have reduced acetate formation [34]. Nevertheless, we continued to work with strains that carried an *iclR* deletion to assure complete derepression of the glyoxylate shunt. Deletion of *ackA* (Sy510) increased malate production by 24% and decreased acetate (Table 2). Deleting *poxB* alone (Sy508) or together with *ackA* (Sy512) strongly reduced acetate production, but also resulted in an up to 50% drop of malate production compared to strain Sy510, which was mainly due to an augmented production of pyruvate (Table 2). These results showed that the major metabolic by-product acetate could not be channeled into the Krebs cycle by simply deleting the *ackA* and *poxB*-dependent acetate pathways. In addition, the strains that carried the *ackA* deletion alone or in combination with a deletion of *poxB* (Sy510, Sy512) exhibited strongly extended lag phases (not shown) which was in agreement with previous studies [35, 36]. The strategy of increasing malate production by directly engineering the acetate pathways was therefore dismissed.

To identify alternative metabolic targets to increase malate production without deleting the acetate pathways we carried out a carbon flux analysis on strain Sy504. The strain was cultivated on ^13^C-labelled glucose in a bioreactor where glucose consumption and the formation of the metabolic end-products biomass, carbon dioxide, and organic acids were monitored. Samples for the quantification of ^13^C-label enrichment in individual metabolite pools were withdrawn manually during exponential phase and quantified by IC-MS/MS analysis [37]. In addition, the label incorporation into acetate and malate was measured using ^1^H 1D-NMR.

**Fig. 2:**
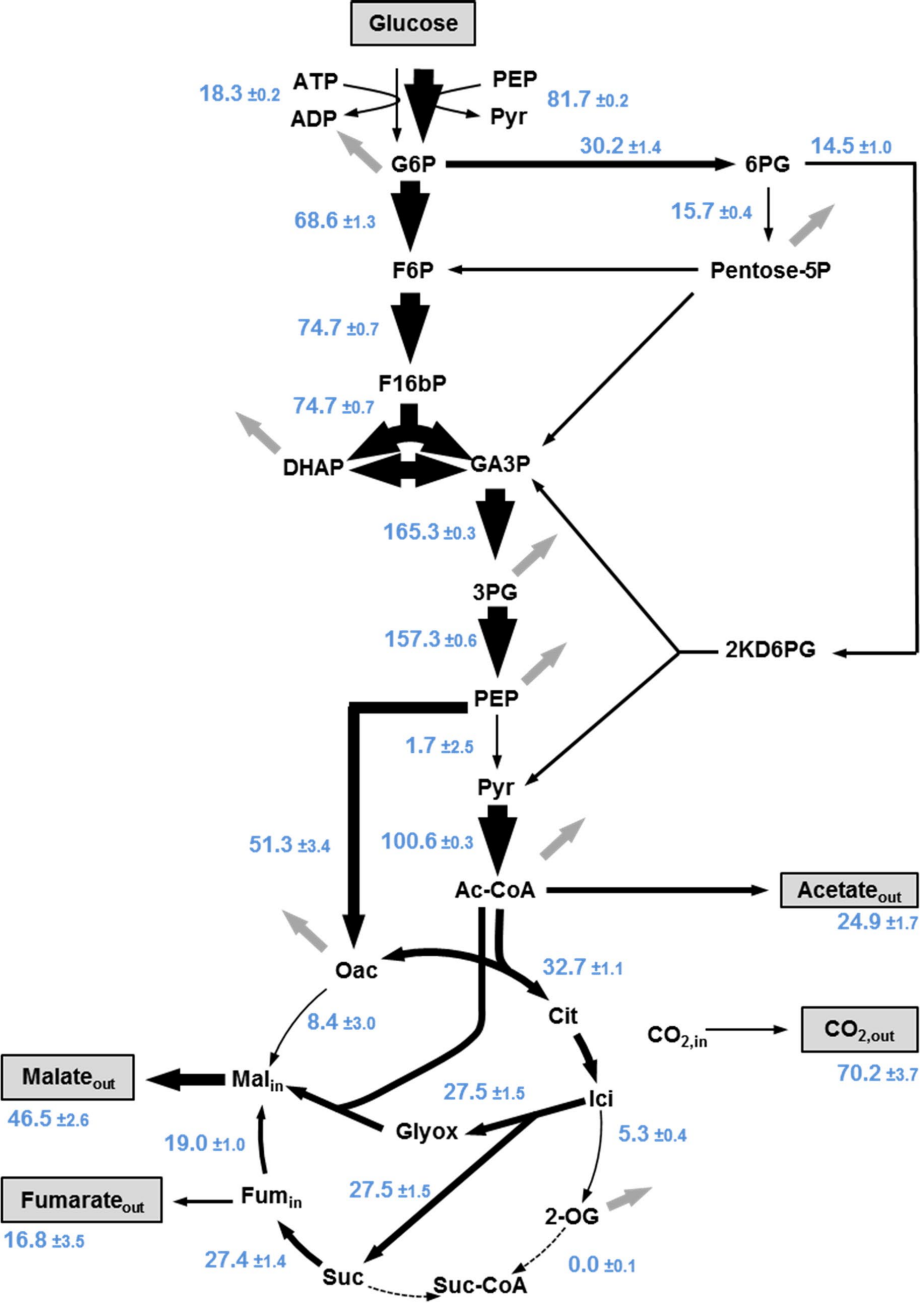
Carbon flux distribution in strain *E. coli Δmdh Δmqo ΔmaeA ΔmaeB ΔiclR*. Fluxes are indicated in blue as mol percent per consumed glucose. Thickness of the arrows corresponds to amount of carbon flux. Boxed metabolites were directly measured. Grey arrows indicate carbon flux into biomass. The flux values were inferred from two independent experiments (*BM* biomass, *G6P* glucose-6P, *F6P* fructose-6P, *F16bP* fructose-1,6-bisP, *DHAP* dihydroxyacetonephosphate, *GA3P* glyceraldehyde-3P, *3PG* 3P-glycerate, *PEP* phosphoenolpyruvate, *Ac-CoA* acetyl-CoA, *Cit* citrate, *Ici* isocitrate, *2-OG* 2-oxoglutarate, *Suc-CoA* succinyl-CoA, *Fum*_*in*_ intracellular fumarate, *Mal*_*in*_ intracellular malate, *6PG* 6P-gluconate, *Ribu5P* ribulose-5P)

The carbon flux distribution in strain Sy504 was calculated from these data and is depicted in Fig. 2. In qualitative agreement to the shake flask experiments we found that malate and fumarate were co-secreted into the supernatant and that acetate was the major metabolic by-product in addition to the two C4 compounds. Unexpectedly, we calculated a direct flux from oxaloacetate towards malate of ~ 8 mol%. This result is hard to explain considering that (i) under aerobic conditions this flux normally occurs in the opposite direction, and (ii) malate dehydrogenase was deleted in the analysed strain. A possible explanation for this putative flux is that carbon flux actually passed from oxaloacetate to fumarate and further to malate through aspartate transaminase, aspartase, and fumarase, respectively.

As expected, strong differences in carbon flux repartitioning between wild-type strains [34, 38, 39] and the malate-producing strain Sy504 have been observed regarding the metabolic fate of PEP and the solicitation of the Krebs cycle and glyoxylate shunt reactions. Since oxaloacetate cannot be regenerated from malate in strain Sy504, the net-flux from PEP to oxaloacetate, that is, the flux over PEP carboxylase minus the flux over PEP carboxykinase, is on average 60% higher in this mutant than in the corresponding *E. coli* MG1655 wild-type strains (Additional file 1: Table S2).

While the flux from pyruvate to acetyl-CoA remained largely the same in all strains, acetyl-CoA was utilized very differently in our strain when compared to wild-type cells. Sy504 produced nearly 50% less acetate, and condensed a particularly large fraction of 30 mol% acetyl-CoA with glyoxylate to yield malate. Even in an Δ*iclR* single mutant, which has a derepressed glyoxylate shunt [34], nearly two-times less acetyl-CoA is directly converted into malate which indicates a particularly strong solicitation of the glyoxylate shunt in the malate-producing strain. The split ratio of fluxes at the isocitrate lyase (Icl)–isocitrate dehydrogenase (Icd) bifurcation was 5 in strain Sy504 (Additional file 1: Table S2), whereas it was only 0.5 in the Δ*iclR* mutant [34] during growth on glucose, and 0.4 during growth of wild type cells on acetate [40]. Even more strikingly, no carbon flux was observed between 2-oxoglutarate and succinate in our malate-producing strain (Fig. 2). Our study did not identify the actual physiological reason for this unexpected behaviour. However, since high flux across the glyoxylate shunt is required to attain the maximum theoretical malate yield of 1.33 mol/mol with the this pathway [9], this result of the flux analysis made clear that malate production could not be significantly increased by engineering the Icl/Icd node.

Instead, our attention was attracted by the observation that strain Sy504 produced 50% less acetate and 20% more malate in the bioreactor when compared to shake flask cultivations (Table 1, Fig. 2). We hypothesized that better aeration of the fermenter cultures could explain increased malate production in the bioreactor. Oxygen limitation is known to cause both ArcA-mediated repression of genes implicated in respiratory metabolism and accumulation of NADH [41]. We therefore tested the effect of deleting the transcriptional repressor ArcA, and of overexpressing the NADH-insensitive citrate synthase mutant GltA^R164L^ [42]. We found that the deletion of *arcA* in strain Sy729 which bears the *iclR* deletion increased the malate yield to 0.53 mol/mol (Table 2). The additional overexpression of GltA_R164L_ in strain Sy731 resulted in a further improved malate yield which amounted to 0.82 mol/mol. It is of note that this increase of malate yield with strain Sy731 was accompanied by a strongly reduced acetate production which dropped to only 0.28 mol/mol without requiring the deletion of any of the acetate-producing pathways. The additional deletion of aspartase, encoded by *aspA*, did not increase malate production (not shown).

### The dicarboxylic acid transporter DcuA is the major malate-exporting permease in the engineered strain

**Fig. 3:**
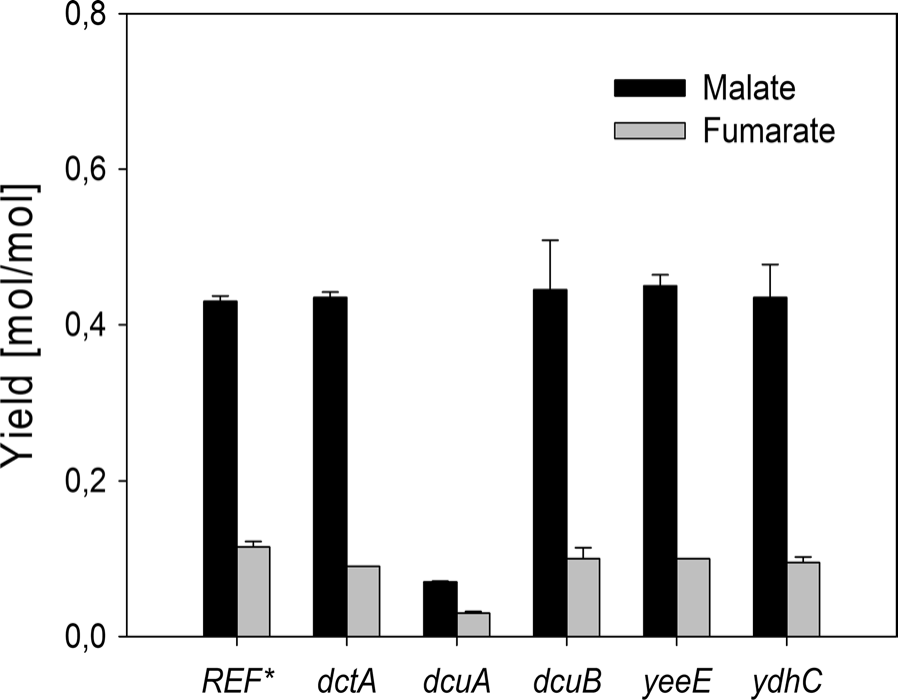
Identification of the major malate-exporting permease. Candidate permeases were individually deleted in parent strain *E. coli Δmdh Δmqo ΔackA*-*pta ΔmaeA ΔmaeB expressing* pACT3w-*ppc*_K620S_ (Sy280 = REF)

Previous studies have shown that malate production could be strongly increased by overexpressing malate-exporting permeases [6, 9]. Alternatively, the deletion of malate permease(s) increases intracellular availability of malate which can be expected to be beneficial for the conversion of malate into other value-added products, such as 2,4-dihydroxybutyric acid [19]. We therefore set out to identify the malate transporter in the malate-producing strain *E. coli Δmdh Δmqo ΔackA*-*pta ΔmaeA ΔmaeB* pACT3w-ppc_K620S_ (Sy280) by deleting the permease DctA, which is required for the uptake of dicarboxylic acids aerobic conditions [43, 44], and the permeases DcuA and DcuB which are responsible for the uptake of dicarboxylic acids under anaerobic conditions [45]. In addition, we tested the impact of deleting two putative transporter proteins, YeeE and YqhC [46], which were found transcriptionally up-regulated in our malate-producing strain (data not shown). We observed that malate and fumarate secretion decreased by ~ 80% upon deletion of the constitutively expressed dicarboxylic acid transporter, DcuA [47], whereas the deletion of the other permeases did not cause any decrease of malate secretion. Thus, DcuA was identified as the major malate-exporting permease under aerobic conditions (Fig. 3). However, plasmid-born overexpression of this transporter did not increase malate production (data not shown).

## Discussion

In the present study we investigated the metabolic requirements for Krebs cycle-dependent production of malic acid in *E. coli.* We found that significant malate production under these conditions required deletion of all malic enzymes and dehydrogenases, and concomitant expression of a malate-insensitive PEP carboxylase. Interestingly, there was almost no gradual increase of malate production upon step-wise introduction of these metabolic modifications, but all genetic modifications had to be present simultaneously to trigger malate production. These results appear to be at variance with data of [48] who reported aerobic production of malate at a yield of 0.56 mol/mol upon expression of a PEP carboxykinase (Pck) from *Mannheimia succiniciproducens* in an *E. coli pta* mutant. It is not clear to us how aerobic malate production could be achieved in this metabolic set-up given that (i) reduction of oxaloacetate (OAA) to malate requires high NADH/NAD ratios which are commonly only achieved under oxygen-limited or anaerobic conditions in *E. coli*, (ii) Pck-dependent synthesis of OAA from PEP requires high carbon dioxide and PEP concentrations [11], which are commonly achieved by imposing either anaerobic conditions or sparging with carbon dioxide [11, 15] and by deleting the PEP-consuming phosphotransferase system or inactivating pyruvate kinase [11, 15, 16]. In addition, (iii) our own results show in agreement with the observations made by Zhang et al. [14] that activity of the malic enzymes prevents significant malate accumulation (Additional file 1: Table S2). The actual reason for these apparent discrepancies remains unclear which may indicate our still incomplete understanding of the metabolic regulation that occurs at the PEP/OAA/malate/pyruvate node in *E. coli*.

The major metabolic by-product in our aerobic metabolic setup was acetate. Our attempts to reduce acetate secretion by deleting the AckA and/or PoxB-dependent pathways came at the cost of extended lag-phases or even reduced malate production. We therefore set out to engineer strains that produced less acetate despite having functional acetate pathways. Acetate production is mainly regulated by citrate synthase activity. Citrate synthase is allosterically inhibited by high intracellular NADH concentrations, and excess acetyl-CoA is converted to acetate instead of being condensed with oxaloacetate to enter the Krebs cycle in the form of citrate [42, 49, 50]. In agreement with this notion, the deletion of the transcriptional repressor of respiratory enzymes, ArcA, and the overexpression of NADH-insensitive citrate synthase reduced acetate formation by 50% and increased malate production by ~ 90% (when comparing strains Sy504 and Sy731). Together, these results indicated that reducing acetate production via engineering NADH metabolism and citrate synthase was an effective means to improve the performance of malate-producing strains.

We found that malate production by our strains coincided with the accumulation of significant amounts of fumarate. Both compounds accumulated in the supernatant at a molar ratio of approximately 8, which is very similar to the ratio of their intracellular concentrations that was estimated with approximately 12 [51]. Thus, because the malate/fumarate ratio is imposed by the thermodynamic equilibrium of the reversible fumarase reaction, Krebs cycle-dependent malate production will almost inevitably coincide with the formation of fumarate as a co-product. In line with these arguments, we found that the dicarboxylic acid transporter DcuA, which accepts both malate and fumarate as substrates [45], was responsible for malate secretion. Only replacement of this rather unspecific permease by a highly malate-specific transporter could reduce fumarate secretion during Krebs cycle-dependent malate production.

In summary, Krebs-cycle dependent malate production can be achieved at the cost of lower maximum product yields and significant accumulation of fumarate as a metabolic by-product. These results make this metabolic engineering strategy unattractive when compared to Ppc or Pck-dependent pathways if malate is the actual target molecule. However, if malate is an intermediate product which is further processed to yield derivatives such as DHB [19], fumarate will not accumulate and the additional cofactors NAD(P)H and ATP which are generated in the Krebs cycle and respiratory chain can be used to increase product yields. Our study has laid the foundation for these microbial product syntheses.

Our study was carried out in aerobic shake flasks on mineral M9 medium. These cultivation conditions are clearly not representative for a fermentation process which enables cost efficient production of malate which would require to carry out the fermentations in bioreactors using low-cost mineral salt medium [52]. Under such conditions, product titers and yields obtained with the strains described in this work may significantly change. However, at the present stage of our analyses we focused on the fundamental genetic requirements for Krebs cycle-dependent malate production, which represents an intermediate stage on the way to the construction of production strains for biosynthesis of malate-derived value-added chemicals such as DHB.

## Additional file

**Additional file 1: Table S1.** Fermentation products of *E. coli* MG1655 mutants in mineral medium with glucose as the carbon source. **Table S2.** Comparison of carbon fluxes given in mol% relative to glucose uptake measured in different studies for wild-type and mutant *E. coli* strains.
