# Supplementary material for: Engineering of Escherichia coli for Krebs cycle-dependent production of malic acid
Source: Microb Cell Fact. 2018 Jul 16;17:113. doi: 10.1186/s12934-018-0959-y (PMC6048880; doi:10.1186/s12934-018-0959-y)
Supplement: Supplementary file 1 — Additional file 1: Table S1. Fermentation products of E. coli MG1655 mutants in mineral medium with glucose as the carbon source. Table S2. Comparison of carbon fluxes given in mol% relative to glucose uptake measured in different studies for wild-type and mutant E. coli strains. [file 12934_2018_959_MOESM1_ESM.docx]

**Table S1**: Fermentation products of *E. coli* MG1655 mutants in mineral medium with glucose as the carbon source

| Strain | Genotype | Plasmid | Cells | Malate | Fumarate | Pyruvate | Acetate |
| --- | --- | --- | --- | --- | --- | --- | --- |
|  |  |  | [g/g] | [mol/mol] | [mol/mol] | [mol/mol] | [mol/mol] |
| Sy242 | *Δmdh Δmqo ΔackA-pta* | pACT3w-ppc_K620S_ | 0.19 | 0.04 | 0.00 | 0.98 | 0.49 |
| Sy254 | *Δmdh Δmqo ΔackA-pta ΔmaeA* | pACT3w-ppc_K620S_ | 0.31 | 0.09 | 0.08 | 0.28 | 0.32 |
| Sy252 | *Δmdh Δmqo ΔackA-pta ΔmaeB* | pACT3w-ppc_K620S_ | 0.29 | 0.14 | 0.05 | 0.60 | 0.31 |

**Table S2**: Comparison of carbon fluxes given in mol% relative to glucose uptake measured in different studies for wild-type and mutant *E. coli* strains

|  | Sauer^1^ | Nicolas^2^ | Waegeman^3^ | our study | |
| --- | --- | --- | --- | --- | --- |
| Fluxes | Wild type | Wild type | Wild type | Δ*iclR* | Sy504 |
|  | [Mol%] | | | | |
| (PEP to OAA) – (OAA to PEP) | 34 | 33 | 30 | 19 | 51 |
| pyruvate to acetyl-CoA | 93 | 99 | 109 | 116 | 100 |
| acetyl-CoA into citrate/acetate/malate/BM | 30/42/0/21 | 25/48/0/26 | 43/49/0/17 | 53/26/17/20 | 33/25/28/15 |
| isocitrate into  glyoxylate/2-oxoglutarate | 0/30 | 0/25 | 0/43 | 17/36 | 27/5 |

All strains were cultivated in batch experiments with glucose excess. BM - biomass.

^1^Sauer U, Canonaco F, Heri S, Perrenoud A, Fischer E. The soluble and membrane-bound transhydrogenases UdhA and PntAB have divergent functions in NADPH metabolism of Escherichia coli. J Biol Chem. 2004;279:6613–9.

^2^Nicolas C, Kiefer P, Letisse F, Krömer J, Massou S, Soucaille P, et al. Response of the central metabolism of Escherichia coli to modified expression of the gene encoding the glucose-6-phosphate dehydrogenase. FEBS Lett. 2007;581:3771–6.

^3^Waegeman H, Beauprez J, Moens H, Maertens J, De Mey M, Foulquié-Moreno MR, et al. Effect of iclR and arcA knockouts on biomass formation and metabolic fluxes in Escherichia coli K12 and its implications on understanding the metabolism of Escherichia coli BL21 (DE3). BMC Microbiol. 2011;11:70.
